# Stigma Associated with Mental Illness: Perspectives of Judges and Lawyers in Lebanon

**DOI:** 10.1192/j.eurpsy.2024.1205

**Published:** 2024-08-27

**Authors:** R. Mroue, M. Cherro, G. Kassir, N. Dandan, E. Ghossoub

**Affiliations:** ^1^Faculty of Medicine, American University of Beirut; ^2^Department of Psychiatry, American University of Beirut Medical Center, Beirut, Lebanon

## Abstract

**Introduction:**

Legal professionals frequently encounter forensic mental health issues in the criminal justice system. These issues can significantly impact the outcome of cases, making it essential to understand the attitudes and perceptions of these experts towards mental illness. Despite a high number of individuals with mental illness in prisons, the availability of forensic mental health services is limited. While prior research has shown widespread stigma towards mental illness, there hasn’t been a study assessing the attitudes of judges and lawyers.

**Objectives:**

This study aims to investigate the stigma related to mental health among Lebanese legal professionals.

**Methods:**

An online questionnaire was sent to judges and lawyers practicing in Lebanon. The survey included a section on demographics and personal data with the following scales: Reported and intended behavior scale (RIBS) which measures mental health stigma–related behavior and Perceived devaluation and discrimination scale (PDD) measuring the extent to which a person believes that most people will devalue or discriminate against someone with a mental illness.

**Results:**

A total of 215 participants, with a mean age of 38.69 and a mean 13.16 years of experience, completed the questionnaire. Most were female (62.8%) and worked as civil attorneys (47.4%). Only a minority received instruction on mental health or mental health law during training (10.7% and 8.8%). About a quarter believed their education on mental health issues was sufficient (27%). Participants with positive attitudes (RIBS) were more likely to have a family member with a mental illness (p value = .001), feel comfortable handling cases involving mental health (p value = .001), and have lived with someone with a mental illness (p value = .007). Feeling adequately educated about mental health issues was associated with lower perceived stigma (PDDS, p value = .021). No significant associations with stigma scores were found for factors like age, gender, occupation, years of experience, contact with a mental health professional, taking psychotropic medications, disclosing personal mental health issues to friends or co-workers, receiving education on mental illness or mental health law, or working with individuals with mental health issues.

**Conclusions:**

The findings imply that enhancing mental health education and awareness within the legal profession could be a key strategy to reduce stigma and improve the overall treatment of individuals with mental health issues within the criminal justice system in Lebanon.

**Disclosure of Interest:**

None Declared

